# Heterotaxy pattern associated with sinus node dysfunction in an adult: A case report

**DOI:** 10.1002/ccr3.7511

**Published:** 2023-06-17

**Authors:** Naman Shah, Sankalp Acharya, Apoorva Tripathi, Himanshi Bisht, Maitri Shah, Aayushi Pareek, Asmita Gera, Abhigan Babu Shrestha, Vikash Jaiswal

**Affiliations:** ^1^ GMERS Medical College Sola Ahmedabad India; ^2^ B. J. Medical College Ahmedabad India; ^3^ RHUS College of Medical Science Jaipur Rajasthan India; ^4^ JCCR Cardiology Research Varanasi India; ^5^ M Abdur Rahim Medical College Dinajpur Bangladesh; ^6^ Larkin Community Hospital South Miami Florida USA

**Keywords:** pathology, polysplenia syndrome, SA node dysfunction

## Abstract

**Key Clinical Message:**

A 26‐year‐old male patient admitted to the hospital ward with experience of repetitive syncopes for a year. The patient was diagnosed with sick sinus syndrome. The aim of this clinical report is to highlight the variability of anatomical findings associated with polysplenia pattern.

**Abstract:**

This case report presents a 26‐year‐old male patient who presented to the medical ward with a complaint of repeating blackouts for a year. The patient was then diagnosed with sick sinus syndrome, and further investigations revealed left isomerism, polysplenia, and no congenital heart defects. Holter monitoring, ultrasonography, electrocardiography, and computed tomography were used to confirm the diagnosis. The patient underwent DDDR pacemaker implantation for the treatment of SA node dysfunction. The report highlights the variability of anatomical findings associated with polysplenia pattern and the various types of heartbeat disruptions that may occur in the atrial appendages of the left side isomerism.

## 1 INTRODUCTION

Heterotaxy pattern, also called Situs ambiguous, is a medical condition characterized by an atypical arrangement of the internal organs in the thorax and abdomen. This disease can lead to complicated congenital deformities that affect various organ systems, such as the circulatory, pulmonary, digestive, reproductive, and the urinary system. It often causes serious health problems. It can alter the structure of the heart, including the attachment of the large blood vessels that carry blood to and from the rest of the body. Pulmonary system is also impacted, lungs affected tend to have different numbers of lobes than normal anatomical lungs, and the size of bronchi is also affected. The condition also varies in severity. Some individuals may experience mild health problems while others may face potentially life‐threatening risks even with treatment during infancy or childhood.^1^

The mortality rate for most patients diagnosed with polysplenia syndrome is alarmingly high, with many not surviving beyond the age of five. This is largely attributed to the presence of severe cardiac abnormalities. Yet, a miniscule fraction (5.0%–10.0%) of people with this syndrome have normal heart or minor cardiac abnormalities and can mature without exhibiting any significant signs and symptoms. This case presentation is an example of such an occurrence.^2^

Heterotaxy syndrome is classified into two main categories: right isomerism and left isomerism. Right isomerism mainly includes septal defects in the heart. Both versions are correlated with complicated heart malfunctions.^3^ Hereby, we report sick sinus syndrome in a 26‐year‐old man who was admitted to our clinic with complaints of syncope (sick sinus syndrome) and was found to have left isomerism on further investigation. The aim of this discussion was to explore the various heart rhythm disturbances that individuals with polysplenia syndrome may experience, the genetic mutations associated with the syndrome, and the management of sinus node dysfunction in such patients.

## 2 CASE PRESENTATION

**FIGURE 1:**
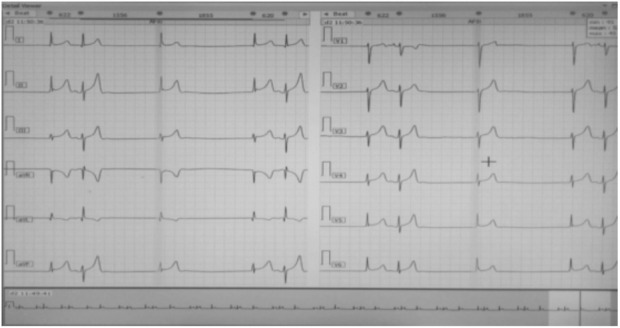
Electrocardiography shows sinus node arrest with a junctional escape at 35–40 bpm.

**FIGURE 2:**
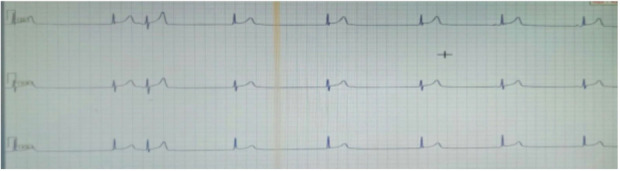
Holter shows sinus pause and sinus arrest with junctional escape beats/rhythm, confirming the diagnosis of sick sinus syndrome).

A 26‐year‐old male patient presented to our medical facility with a history of multiple episodes of fainting occurring throughout the previous year. Upon further inquiry, the patient mentioned that he had experienced three episodes of fainting within a period of 1 year. On examination, his pulse was at 40/min with an irregular rhythm. His blood pressure (BP) reading registered as 140/90 mmHg. The cardiovascular system examination showed no abnormalities. Further investigations showed that the full blood count and sugar levels were in the normal range. Additionally, the kidney function tests, serum electrolytes, and liver tests all yielded normal results. The electrocardiography (ECG) results showed the presence of sinus node arrest accompanied by a junctional escape that was recorded at a rate of 35–40 beats per minute (Figure 1). Holter monitoring was performed to verify the diagnosis and identify any presence of AV blockage or alternate rhythm disturbances that might result in syncope. The test results confirmed sick sinus syndrome, which showed pauses and arrest in sinus rhythm along with junctional escape beats/rhythm (Figure 2). An ultrasonography (USG) revealed a midline liver that was more toward the left side, with a leftward hilum and normal hepatic veins, and inferior vena cava on the right side. No spleen on the left side was identified on USG, consistent with situs ambiguous abdominis.

**FIGURE 3:**
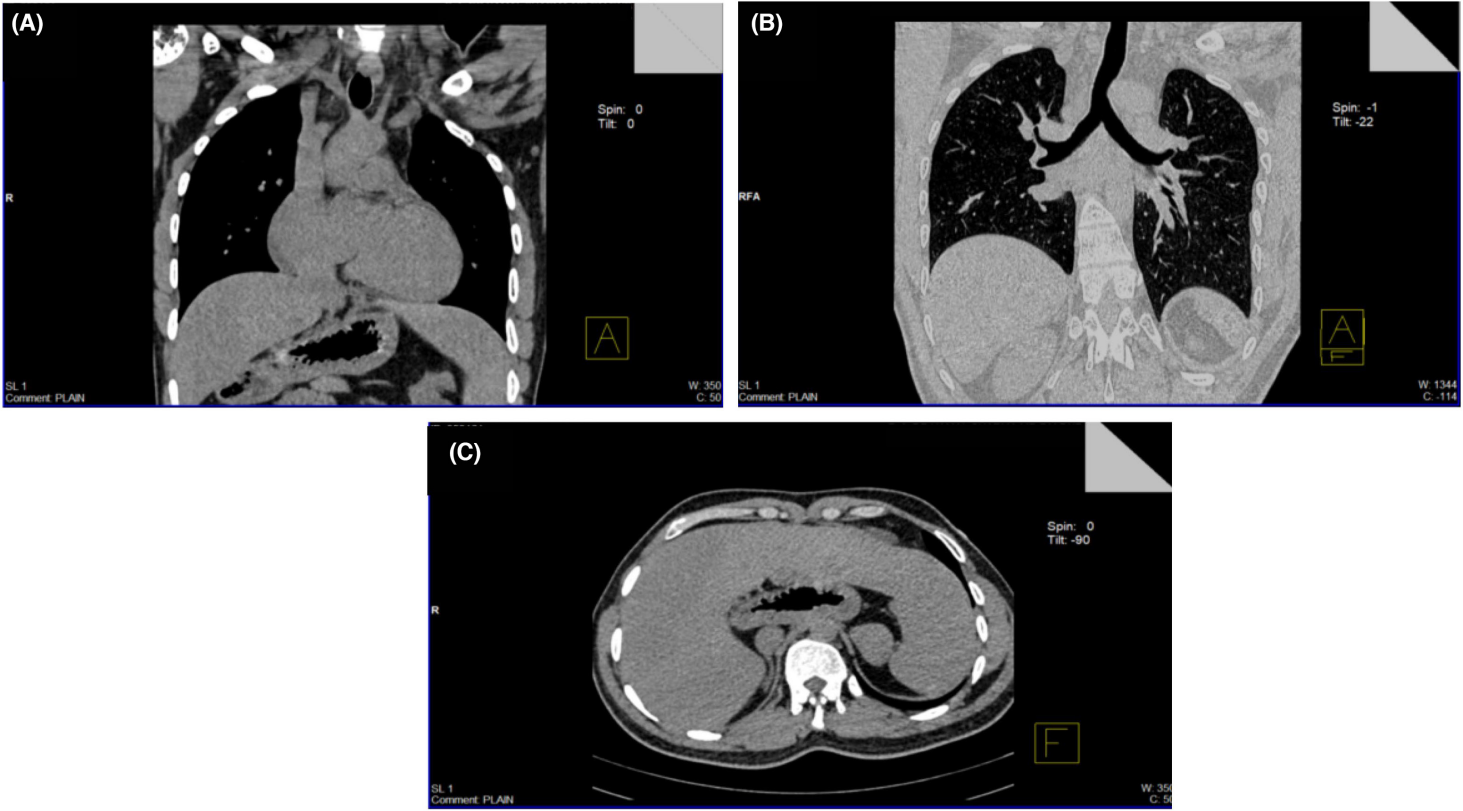
(A) CT scan (HRCT) of the chest with upper abdomen shows evidence of liver on the left side of the abdomen and midline stomach. (B) CT scan of the chest (HRCT) with upper abdomen shows bilateral trilobed lung with hyparterial bronchus. (C) CT scan on axial view shows evidence of liver on the left side of the abdomen and midline stomach. Multiple accessory spleens (at least 7) are seen in the abdomen.

Echocardiography revealed normal left ventricular ejection fraction and no congenital heart defects. An HRCT chest with upper abdomen was conducted on a 128‐slice dual source dual energy scanner. The scan results revealed the presence of liver on the left side of the abdomen and in the midline stomach (Figure 3A). The abdomen contained numerous accessory spleens, at least seven. A bilateral trilobed lung with hyparterial bronchus is seen (Figure 3B). Situs ambiguous with polysplenia syndrome was diagnosed on CT (Figure 3C). The patient received a dual‐pacing dual‐sensing dual‐response rate‐adaptive (DDDR) pacemaker for sinus node dysfunction. The patient responded well, with improvement in symptoms and findings at follow‐up.

## 3 DISCUSSION

Polysplenia is a syndrome that is characterized by various anatomical findings, and it can be quite complex to manage. In left isomerism, one can expect to find left atrial isomerism with bilateral morphologic left atrial appendages, viscerocardiac heterotaxy, and multiple cardiac malformations such as congenital heart block, atrioventricular septal defects and pulmonary stenosis, multiple polysplenia, bilobed lungs on the left side with hyparterial bronchi, intestinal malrotation, non‐random genitourinary malformations, and an interruption of the inferior vena cava (IVC) accompanied by azygos continuation.

Furthermore, it is also possible for this medical condition to lead to absence of spleen or the condition may result in the formation of numerous small and inefficient spleens (polysplenia) in the abdomen. The liver is located in the center of the body and not in its usual spot on the right side of the abdomen. In cases where there is isomerism of the left atrial appendages, individuals may experience one of three types of heart rhythm disturbances. The heart rhythm disturbances associated with left‐sided isomerism include atrioventricular block, dual atrioventricular nodal pathways, and sinus node dysfunction.^1^, ^3^, ^4^, ^5^ Patients with polysplenia syndrome have been found to exhibit different mutations, such as LEFTYA3, Z1C3.^6^ Our patient, on the other hand, did not exhibit or undergo any study for such mutations.

The reported patient was an adult who had been diagnosed with polysplenia syndrome, had a structurally normal heart, and suffered from sinus node dysfunction. It is worth noting that the occurrence of isolated conduction disorder and a structurally normal heart is quite rare and unusual in patients with polysplenia syndrome.^5^

In patients with sinus node dysfunction, implantations of AAI C/R and DDD C/R pacemakers for electrical stimulation have shown to yield better outcomes due to the reduced incidence of atrial fibrillation, thromboembolic events, and heart failure. This is supported by research findings mentioned in the source.^3^

IVC interruption with azygous continuation is seen in left isomerism which in turn affects the course of a catheter or thrombolytic predictive instrument (TPI) lead while performing temporary pacemaker implantation from femoral vein access. Alternatively, one can choose for internal jugular vein access for TPI.

While performing TPI, one must be aware of the presence of left‐sided SVC and pacemaker lead course accordingly.

## 4 CONCLUSION

In view of the above, it is safe to say that polysplenia syndrome is a complicated condition that can lead to various defective anatomical findings and heart rhythm disturbances. It is rarity that sinus node dysfunction can present with a structurally normal heart as seen in our case. In such cases, using AAI C/R and DDD C/R pacemakers are the sole management strategy. However, there are certain intricate details that have to be paid heed to. Such as, the interruption of IVC with azygous continuation can affect the course of catheter or TPI lead during temporary pacemaker implantation from femoral vein access. In such cases, internal jugular vein access may be preferred. Moreover, the genetic mutations associated with polysplenia syndrome and their impact on patients are untapped research territory that needs to be explored in order to deal with the syndrome in a much more efficient manner.

## AUTHOR CONTRIBUTIONS

**Naman Shah:** Writing – original draft; writing – review and editing. **Sankalp Acharya:** Visualization; writing – review and editing. **Apporva Tripathi:** Writing – original draft; writing – review and editing. **Himanshi Bisht:** Writing – original draft; writing – review and editing. **Maitri Shah:** Writing – original draft. **Aayushi Pareek:** Writing – review and editing. **Asmita Gera:** Writing – original draft; writing – review and editing. **Abhigan Babu Shrestha:** Writing – original draft; writing – review and editing. **Vikash Jaiswal:** Supervision; writing – review and editing.

## FUNDING INFORMATION

None.

## CONFLICT OF INTEREST STATEMENT

None.

## CONSENT

Written informed consent was obtained from the patient to publish this case report and accompanying images. A copy of the written consent is available for review by the Editor‐in‐Chief of this journal on request.

## Data Availability

The authors confirm that the data supporting the findings of this study are available within the article.
